# Bioprospecting of Five *Ocimum* sp. Cultivars from Croatia: New Potential for Dietary and Dermatological Application with Embryotoxicity Tests

**DOI:** 10.3390/ph16070981

**Published:** 2023-07-08

**Authors:** Marija Baković, Lucija Perković, Gabrijela Matijević, Ana Martić, Tamara Vujović, Sara Ekić, Monika Fumić, Sara Jurić, Rozelindra Čož-Rakovac, Marin Roje, Stela Jokić, Igor Jerković

**Affiliations:** 1 Laboratory for Aquaculture Biotechnology, Division of Materials Chemistry, Ruđer Bošković Institute, Bijenička cesta 54, 10000 Zagreb, Croatia; marija.bakovic@irb.hr (M.B.); lucija.perkovic@irb.hr (L.P.); gabrijela.matijevic@irb.hr (G.M.); ana.martic@irb.hr (A.M.); tamara.vujovic@irb.hr (T.V.); rozelindra.coz-rakovac@irb.hr (R.Č.-R.); 2 Laboratory for Chiral Technologies, Division of Organic Chemistry, Ruđer Bošković Institute, Bijenička cesta 54, 10000 Zagreb, Croatia; sara.ekic@irb.hr (S.E.); monika.fumic@irb.hr (M.F.); sara.juric@irb.hr (S.J.); mroje@irb.hr (M.R.); 3 Department of Process Engineering, Faculty of Food Technology, Josip Juraj Strossmayer University of Osijek, Franje Kuhača 18, 31000 Osijek, Croatia; sjokic@ptfos.hr; 4 Department of Organic Chemistry, Faculty of Chemistry and Technology, University of Split, Ruđera Boškovića 35, 21000 Split, Croatia

**Keywords:** basil chemotypes, ethanolic extracts, polyphenol content, biological activities

## Abstract

*Ocimum basilicum* L. is the most common *Ocimum* species, and it is used as an ornamental plant and in food condiments. This unique study examined the chemical composition and biological activities of six extracts from five basil cultivars, including their antimicrobial, antidiabetic, antilipidemic, neuroprotective, and anticollagenase activity. Moreover, their toxicological effects were studied using the zebrafish *Danio rerio*. Volatile components were determined using HS-SPME and GC-MS, while total polyphenols were detected using HPLC and the spectrophotometric Folin–Ciocalteu method. Spectrophotometric assays (DPPH, ABTS, ORAC, FRAP) were performed to determine antioxidant activity, collagenase inhibition, acetylcholinesterase inhibition, and pancreatic lipase inhibition. Antimicrobial activity was determined using the broth microdilution test. The study found that the biological activities of different basil cultivars varied depending on the proportion of active compounds, as determined by chemical analyses. All six basil extracts significantly inhibited α-amylase, while Purple basil extract most significantly inhibited the activity of collagenase, acetylcholinesterase, and pancreatic lipase. Purple basil and Dark Opal basil I extracts exhibited the highest antimicrobial activity, while the Dark Opal basil II extract had the most significant antioxidant potential. The findings in this study suggest that ethanolic basil extracts have the potential to be used as dietary drugs and implemented in antiaging products. This study is unique in its aims to compare the chemical composition and biological activities of basil cultivars from Croatia and to evaluate potential toxicological effects through embryotoxicity tests on zebrafish *Danio rerio* embryos, and it reports the first evidence of anticollagenase, antidiabetic, and antilipidemic activities for these cultivars.

## 1. Introduction

Approximately 150 *Ocimum* species for medicinal purposes have been documented since ancient times [1,2]. The varying colors of the flowers, leaves, and stems, as well as the plant’s shape, chemical composition, and growth characteristics, distinguish different species [3], with *Ocimum basilicum* being the most common one. Basil is widely used as a food condiment and is also gaining recognition for its potential as alternative medicine [4]. In general, medicinal plants have long been used in healthcare systems, with 80% of the population in developing countries using them for primary healthcare. Due to their low cost, they have become increasingly popular as part of an integrative health practice approach [5]. *Ocimum basilicum* is used to treat skin infections, worms, diarrhea, and headaches [6]. Basil is also used for minor nervous disorders and to relieve rheumatic pain. The dried leaves are said to be a remedy for nervous headaches [7], and it has been shown that there is a direct effect of basil constituents on the central nervous system. For example, ethanolic extracts obtained from *O. sanctum* leaves increases the activity of acetylcholinesterase in the cerebral cortex, striatum, hypothalamus, and hippocampus of the brain [8], probably through the action of antioxidant activity [9]. Basil cultivars have the genetic capacity to produce a variety of chemical compounds, resulting in a variety of chemotypes within the same species [10]. The qualitative and quantitative compositions of essential oils in different basil cultivars have also been shown to differ [10]. In Gayer et al.’s (1996) research, the presence and concentration of the major essential oil components varied significantly between the different accessions, varying from being absent in some genotypes to making up more than 90% of the total essential oil composition in others.

Although numerous research groups have examined *O. basilicum* bioactivities (e.g., anticancer activity, radioprotective activity, antimicrobial activity, anti-inflammatory effects, immunomodulatory activity, antistress activity, antidiabetic activity, antipyretic activity, antiarthritic activity, antioxidant activity, as a prophylactic agent, and in cardiovascular disease) [3,11,12,13,14,15,16], comparative research of different basil varieties is scarce [17,18]. Furthermore, while many studies have explored the bioactivities and methods of basil essential oil (BEO) delivery in food products [10,18,19,20,21,22], information regarding the chemical characterization and biological activity of basil varieties grown in Croatia is still limited. The Croatian MAPs (Medicinal and Aromatic Plants) collection, started in 1998, includes more than 900 accessions of over 180 MAPs. The majority of accessions are made up of Croatian wild material. One of the goals of the Croatian Bank of Plant Genes is the collection of MAP species, and numerous collecting excursions are planned annually. A descriptor list for basil species *(Ocimum* sp.) has been created based on Hiltunen and Holm (1999) [23].

The lack of previously mentioned information led to the present research, which focuses on the cultivars belonging to Genovese, Dark Opal, Purple, Lemon, and Cinnamon basil, all grown in Croatia. The aims of this study were (a) to investigate the headspace composition of the five basil cultivars by headspace solid-phase microextraction (HS-SPME) followed by gas chromatography and mass spectrometry (GC-MS) in order to indicate possible chemotypes and to compare the samples; (b) to determine the phenolic composition of the cultivars using high-performance liquid chromatography (HPLC); (c) to determine the biological activity of extracts of the five cultivars by in vitro spectrophotometric assays (collagenase, acetylcholinesterase, pancreatic lipase, and α-amylase inhibition) as well as their antimicrobial and antioxidant activities; and finally, (d) to determine zebrafish embryotoxicity potential of basil extracts in vivo.

## 2. Results

### 2.1. Chemical Composition of Headspace Volatiles of Five Basil Cultivars

The results containing the headspace composition of basil cultivars are presented in Table 1. In order to obtain more comprehensive results, two fibers were used for HS-SPME: DVB/Carbon WR/PDMS and PDMS/DVB. After conducting GC and GC-MS analyses, 51 compounds were identified in the samples. Great chemical variability among the samples can be noted. Using HS-SPME/GC-MS, it is not possible to confirm the exact basil chemotypes since headspace composition can differ from the composition of the corresponding essential oil.

The Genovese basil type contained linalool (40.82% and 47.83% for I and II, respectively) and eugenol (21.16%; 18.26%) as the major headspace compounds, indicating a possible linalool/eugenol chemotype. Other compounds (listed in Table 1) were sesquiterpene hydrocarbons like germacrene D (3.79%; 4.21%), γ-cadinene (3.78%; 3.55%), cis-α-bergamotene (3.46%; 2.44%) and *trans*-α-bergamotene (3.46%; 2.44%) followed by t-cadinol (2.80%; 3.80%) and 1,8-cineole (2.91%; 1.98%).

The Dark Opal basil type was investigated using two different samples. The first sample most likely belonged to the linalool/eugenol chemotype (linalool 45.77%; 54.60%; eugenol 21.14%; 22.53%), while the second sample indicated linalool chemotype (60.43%; 65.20%) with a minor abundance of eugenol (1.64%; 1.13%), confirming the chemical variability of the Dark Opal basil type. The first sample was characterized by the presence of the sesquiterpenes t-cadinol (4.55%; 3.82%), γ-cadinene (3.50%; 3.00%), and *cis*-α-bergamotene (3.04%; 2.80%), while the second sample was characterized by the monoterpene 1,8-cineole (4.12%; 3.86%) and the sesquiterpenes germacrene D (3.94%; 3.41%) and β-elemene (4.78%; 4.36%).

The Cinnamon basil type was characterized by a high abundance of the oxygenated monoterpenes citral (24.26%; 22.77%), neral (13.12%; 12.13%), and linalool (8.22%; 9.16%), and the sesquiterpenes caryophyllene oxide (5.36%; 5.17%), *trans*-α-bergamotene (6.07%; 6.01%), and *trans*-caryophyllene (4.52%; 4.64%).

The Purple basil type was characterized by a high abundance of estragole (35.28%; 33.70%), linalool (24.75%; 28.82%), and eugenol (11.00%; 11.17%).

The Lemon basil type was characterized by a high abundance of citral (36.27%; 41.47%) and neral (20.57%; 25.54%), indicating citral/neral chemotype. Other less abundant constituents were the sesquiterpenes (*E*)-α-bisabolene (6.38%; 5.26%), *trans*-caryophyllene (5.23%; 4.00%), and *trans*-α-bergamotene (3.10%; 2.51%).

### 2.2. Determination of Phenolic Profile of the Five Basil Cultivars

The phenolic profile of the five different basil cultivars was investigated. Dominant polyphenols identified by HPLC (Table 2) in Genovese, Dark Opal basil II, Purple basil, Cinnamon, and Lemon basil were rosmarinic acid (13.422–24.826%) followed by rutin (2.018–3.789%). The major phenolic compound in Dark Opal basil I was ferulic acid (16.842%), while rosmarinic acid was not detected. This is the only studied species in which rosmarinic acid and rutin were not identified. Other important phenols were detected in small proportions such as vanillic acid; in species Dark Opal basil II, Purple and Cinnamon basil (3.054, 3.439, 3.451%); caffeic acid in Lemon, Dark Opal basil I and Genovese (2.744, 3.185, 3.522%); myricetin in all species with the exception of variety Genovese basil (0.762–2.036%). Diosmin was detected in all species from 0.784% (Purple basil) to 3.473% (Genovese basil). Other phenols were also detected and given in Table 2.

### 2.3. Anticollagenase, Neuroprotective, Antidiabetic, and Antilipidemic Properties

**Figure 1:**
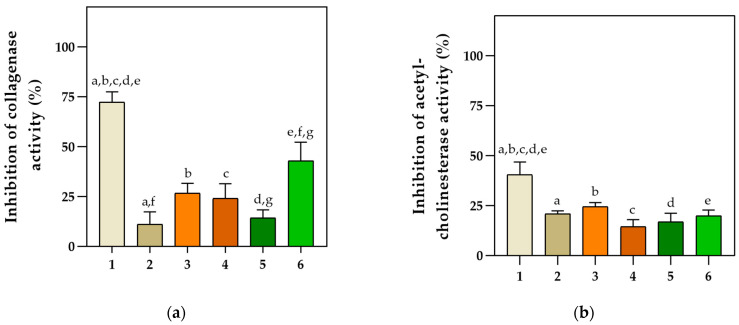

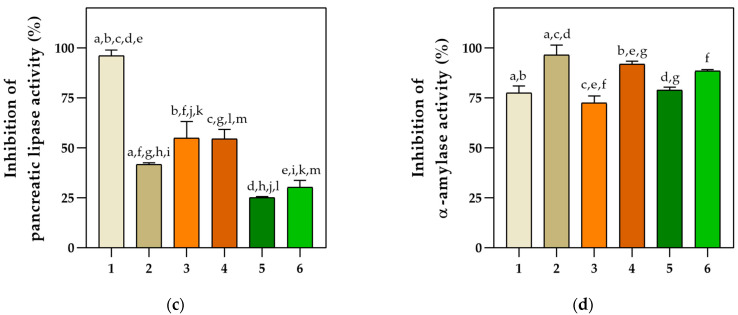
Inhibitory potential of six basil extracts toward four enzymatic activities: (**a**) collagenase, (**b**) acetylcholinesterase, and (**c**) pancreatic lipase, all tested at an extract concentration of 3.3 mg/mL; and (**d**) α-amylase tested at an extract concentration of 0.83 mg/mL. Data are presented as mean ± SD. Common letters indicate a significant difference between the samples (*p* < 0.05). (1—Purple basil, 2—Cinnamon basil, 3—Dark Opal basil I, 4—Lemon basil, 5—Genovese basil, 6—Dark Opal basil II).

Ethanol extracts of the different basil cultivars were evaluated for their ability to inhibit the activity of different enzymes. The tested concentration of 3.3 mg/mL exhibited the highest inhibitory potential against collagenase, acetylcholinesterase, and pancreatic lipase (Figure 1a–c), while the inhibitory activity against α-amylase was most effective at an approximately four times lower concentration of 0.83 mg/mL (Figure 1d).

Among the tested extracts, Purple basil had the most significant inhibitory effect on collagenase (72.42%), acetylcholinesterase (43%), and pancreatic lipase activities (96.21%). However, Cinnamon basil did not show a significant inhibitory effect on collagenase and acetylcholinesterase (<20%), but it expressed the highest α-amylase inhibitory effect (97.44%). Lemon basil and Dark Opal II followed closely, with α-amylase inhibitory effects of 91.91% and 89.32%, respectively. In general, all samples exhibited high inhibitory potential against α-amylase activity. Lemon basil also exhibited a significant inhibitory effect (*p* < 0.05) on pancreatic lipase (54.92%), followed by Dark Opal I (54.54%). Furthermore, Dark Opal II showed the second-highest inhibitory effect on collagenase activity (42.95%).

### 2.4. Antimicrobial Activity of Basil Extracts

In this study, we investigated the antimicrobial activities of extracts from five different basil cultivars. Results showed that extracts from Purple basil and Dark Opal basil I have the most potent activities against the tested bacterial strains (Table 3), while other extracts showed significantly lower effects.

MIC values were obtained for these two extracts, which corresponded to the concentrations of 150 µg/mL (*w*/*v*) for *S. aureus* and 300 µg/mL for *B. subtilis*. In addition, at 300 µg/mL, the MBC was reached for both extracts. Ethanolic extracts also showed partial dose-dependent inhibition of the Gram-negative bacterium *E. coli*, although MIC was not reached for the tested concentrations (300 µg/mL). Basil extracts showed no significant inhibitory effect on other Gram-negative bacteria tested, nor on *C. albicans*.

### 2.5. Antioxidant Activity Determination

**Figure 2:**
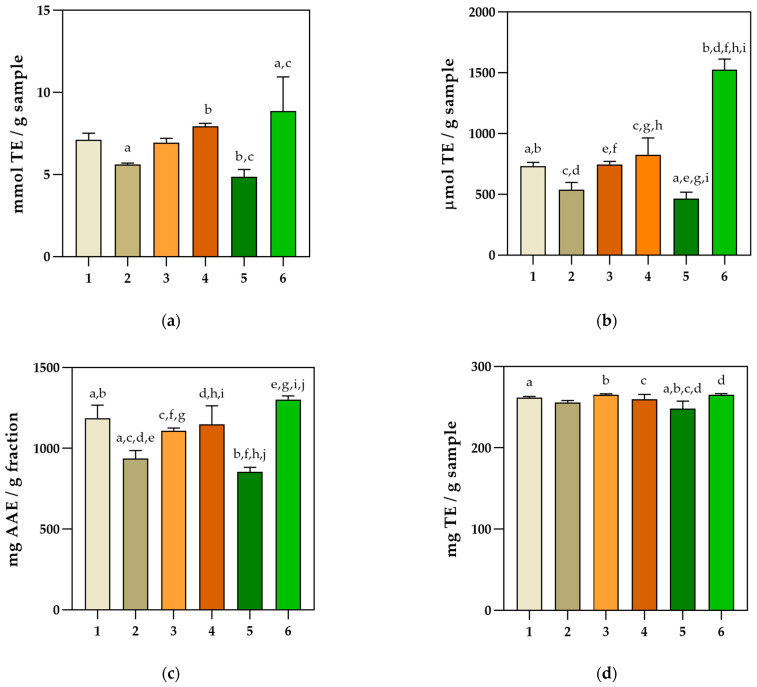
Radical scavenging effect of six basil extracts using (**a**) ferric reducing antioxidant power (FRAP), (**b**) oxygen radical absorbance capacity (ORAC), (**c**) 2,2-diphenyl-1-picryl-hydrazyl (DPPH), and (**d**) reduction of radical cation (ABTS) in vitro assays (mean ± SD; *n* = 3). Common letters indicate a significant difference between the samples (*p* < 0.05). (1—Purple basil, 2—Cinnamon basil, 3—Dark Opal basil I, 4—Lemon basil, 5—Genovese basil, 6—Dark Opal basil II).

**Figure 3:**
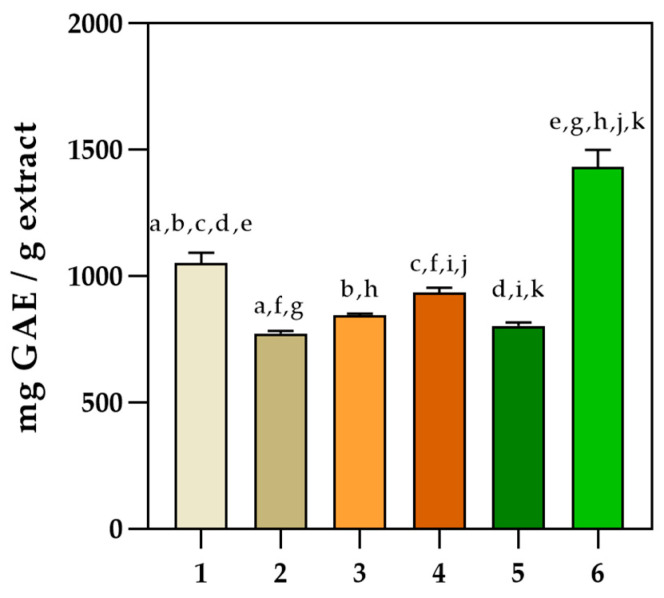
Total phenolic content determination using the Folin–Ciocalteu method (mean ± SD; *n* = 3). Common letters indicate a significant difference between the samples (*p* < 0.05). (1—Purple basil, 2—Cinnamon basil, 3—Dark Opal basil I, 4—Lemon basil, 5—Genovese basil, 6—Dark Opal basil II).

In this research, four spectrophotometric analyses were performed, namely DPPH, ABTS, ORAC, and FRAP assays (Figure 2) [24], while the Folin–Ciocalteu assay was employed to assess the total phenolic content of obtained extracts (Figure 3). Because there are several mechanisms of action and various numbers of antioxidants in such a complex mixture, a single assay cannot accurately reflect the antioxidant activity of a single extract.

The ferric reducing antioxidant power (FRAP) in vitro assay revealed that the Dark Opal basil II sample had the highest activity, followed by Lemon basil and Purple basil (Figure 2a). Significantly higher (*p* < 0.0001) antioxidant activity was observed for the Dark Opal basil II sample compared with other extracts by implementing the ORAC assay (Figure 2b). The DPPH assay confirmed the highest antioxidant activity for Dark Opal basil II (1302.07 ± 21.77 mg AAE /g extract), followed by Lemon basil and Dark Opal basil I, with activities of 1149.65 ± 113.92 mg AAE /g extract and 1109.63 ± 15.52 mg AAE /g extract, respectively (Figure 2c). By implementing ABTS assay, the highest antioxidant activity was also observed for the Dark Opal basil II sample (Figure 2d). The results obtained using the Folin–Ciocalteu method (Figure 3) revealed that the highest total phenolic content was also observed for Dark Opal basil II sample (1431.99 ± 66.73 mg GAE/g extract) followed by Purple basil and Lemon basil.

Multiple variable analysis was used to evaluate the correlation between all the spectrophotometric methods (ABTS, DPPH, ORAC, FRAP, and Folin–Ciocalteu) [25]. Pearson’s correlation coefficient was significantly high between the DPPH, FRAP, and ORAC assays with the Folin–Ciocalteu method and among each other (Table 4). Lower correlations were obtained between the ABTS and the Folin–Ciocalteu method, and the ABTS and ORAC assays.

### 2.6. Embryotoxicity Testing

No negative impact on zebrafish survival was observed during exposure to serial dilutions (0.01–0.33 mg/mL) of the *Ocimum* sp. extracts. The only exception was observed for Purple basil and Cinnamon basil samples at an exposure concentration of 0.33 mg/mL, which caused 90% mortality, while exposure of embryos to lower concentrations caused no mortality. The mortality of individuals in the negative control group in artificial water was not recorded. Developmental abnormalities were observed during exposure to 0.17 mg/mL of both Cinnamon basil and Purple basil extracts; specifically, in 30% and 20% of embryos, respectively. Exposure of embryos to lower tested concentrations (0.08, 0.04, 0.02, 0.01 mg/mL) did not cause any developmental abnormalities. The most common sublethal effects observed in zebrafish embryos recorded after 96 h of exposure to basil extracts were pericardial edema and blood accumulation. Larval hatching was monitored 96 h after fertilization. No differences were recorded compared with the control group.

## 3. Discussion

This research represents the first comprehensive investigation of six samples from five different cultivars of *Ocimum* sp. in terms of their chemical composition and their potential health benefits (antiobesity, antidiabetic, antimicrobial, antioxidative, neuroprotective, and anticollagenase potential). The HPLC method was used to identify the polyphenol composition of the extracts, while headspace extraction followed by GC-MS was used to identify compounds related to the classification of basil chemotypes.

Basil essential oil (BEO) has been extensively studied, and its composition is known to vary depending on the plant’s phenological stage and chemotype [26,27,28]. Different BEO chemotypes have been identified based on the predominant constituents (e.g., linalool, methyl chavicol (estragole), methyl eugenol, eugenol, citral, or α-bergamotene) [26,27,29]. For example, the European chemotype is characterized by high concentrations of linalool and estragole, while the tropical chemotype is rich in *trans*-methyl cinnamate. Additionally, the Reunion chemotype has high concentrations of estragole, while the chemotype from Russia and North Africa are eugenol-rich [30].

High linalool percentage was detected in both of our samples of the Dark Opal basil cultivar, indicating linalool chemotype. This finding is consistent with the previous research on the Dark Opal basil type from Russia [27]. Furthermore, Dark Opal basil II contained a small amount of eugenol which can be linked to the Dark Opal basil type from the United Kingdom and the United States [29]. The results obtained for the Cinnamon basil type did not indicate any specific chemotype, and the volatile profile showed striking differences compared with previous studies on the BEO of this cultivar. In contrast to the basil type from Portugal, where linalool was the predominant volatile compound [17], our research found a high abundance of oxygenated monoterpenes (citral and neral) in the Cinnamon basil type. Similarly, the chemical profile of the Cinnamon basil type collected in Greece showed the oxygenated monoterpenes methyl chavicol, linalool, and germacrene D as the major compounds [31]. It was previously reported that Purple basil has various chemotypes [32], including methyl cinnamate and methyl cinnamate/linalool. However, our research found a different headspace composition indicating a high share of estragole and linalool. Citral isomers were the major compounds detected in the Lemon basil type investigated within this research. The citral-rich chemotype has already been reported as the most common chemotype of *O. citriodorum* [10,33]. However, the methyl chavicol-rich type has also been reported [34], as well as linalool chemotype [17]. Linalool was the predominant compound in the Genovese cultivar from our research, followed by eugenol. Linalool was also the predominant compound in the Genovese basil cultivar from Greece [31] as well as from Croatia, Macedonia, Italy, Slovakia, Austria, Canada, and Germany [27]. It was reported that the samples from the Genovese cultivar from Italy belonged to the eugenol > methyl eugenol chemotype [35].

The composition of essential oils may vary based on environmental factors, genetics, chemotype, and plant nutritional status, and therefore results from different studies may differ. Hussain et al. (2008) claim that the chemical composition fluctuates according to the season, as does the basil essential oil (BEO) content [36]. Linalool, eugenol, and methyl cinnamate, as well as 1,8-cineole, methyl eugenol, geraniol, geranial, neral, and α-bergamotene, were determined as the prevalent BEO components [6,27,37]. These results correspond to compounds detected in our research. Samples of *Ocimum* sp. covered by this research were collected in summer, when sesquiterpene hydrocarbons tend to be prevalent compounds (24.3%), according to the literature [36]. Certain studies have reported that the dried leaves contain the biggest share of oxygenated monoterpenes, while sesquiterpene hydrocarbons are the most preserved in fresh leaves. This could be linked to the nonsignificant amount of sesquiterpene hydrocarbons in most of our samples [38].

In addition to other secondary metabolites, phenolic acids and flavonol glycosides are the main phenolic components in basil [39,40]. Among the phenolic acid compounds identified by HPLC in the six different ethanol extracts of *Ocimum* sp., rosmarinic acid and ferulic acid were found to have the highest area percentages. Among the flavonoids previously identified in *Ocimum* species (quercetin, apigenin, rutin, and luteolin) [41], rutin stands out as having the highest area percentage identified by HPLC. Interestingly, rosmarinic acid was not identified in the Dark Opal basil I ethanol extract, possibly due to a high limit of detection. Rutin and rosmarinic acid have been the focus of many studies investigating the health benefits of basil, highlighting their potential therapeutic properties [42]. The total phenolic content in basil extracts as measured by the spectrophotometric Folin–Ciocalteu method was the highest for Dark Opal basil II, which also exhibited the highest antioxidant activity as evaluated by four in vitro tests. Although belonging to the same cultivar, Dark Opal basil I exhibited approximately 45% lower polyphenolic content. This could be explained by the absence of rosmarinic acid in the Dark Opal basil I extract. By implementing an ABTS assay, the highest antioxidant activity was also observed for the Dark Opal basil II extract, which may be attributed to its high phenolic content (rosmarinic and vanillic acid, rutin, apigenin, epicatechin). Those compounds are responsible for trapping the cation radical ABTS•+ by providing an H+ ion [16]. The ferric reducing antioxidant power (FRAP) in vitro assay confirming the highest activity for the Dark Opal basil II followed by Lemon basil and Purple basil indicates electron transfer as a dominant mechanism of action in those samples. Based on literature reports, antioxidant activity and phenolic content are in strong correlation due to their ability to donate hydrogen, reducing agents, or chelate metals [43]. This connection was also validated in the investigated basil acetone extracts [44]. In our research, multiple variable analysis was used in order to evaluate the correlation between all the spectrophotometric methods used (ABTS, DPPH, ORAC, FRAP, and Folin–Ciocalteu) [24]. Pearson’s correlation coefficient was significantly high between the DPPH, FRAP, and ORAC assays with the Folin–Ciocalteu method and among each other. These correlations suggest that the antioxidant activity of these samples strongly depends on the polyphenolic content [45]. Lower correlations were obtained between the ABTS assay and the Folin–Ciocalteu method, and between the ABTS and ORAC assays, indicating the importance of using multiple antioxidant assays to evaluate complex sample extracts. Different extraction methods and solvents used in the procedure and different culturing conditions affect the polyphenolic content, which may be the reasons for the differences between the results [46,47,48].

Cinnamon basil ethanolic extract with the highest content of detected polyphenols by qualitative HPLC analysis, also exhibited the strongest α-amylase inhibition. Our results are consistent with previously reported findings which suggest that major phenolic constituents (rosmarinic acid and caffeic acid) of methanolic basil extracts and its flavonoids (diosmin, myricetin, epicatechin, quercetin, naringenin), also detected in our extracts, inhibit α-amylase as well as pancreatic lipase [49,50,51,52]. Therefore, *Ocimum basilicum* has been intensively investigated for its antiobesity and antidiabetic activities [53]. In vivo studies conducted on ethanol extracts of basil leaves confirmed a decrease in blood glucose as well as the advanced glycation of end-products in diabetic rats [54]. The study carried out by El-Nahal and Thabet (2012) showed that the main phenolic and flavonoid compounds of ethanolic basil extracts had a positive effect in lowering the weight of hypercholesterolemic rats [55].

Purple basil clearly displayed the highest inhibitory activity toward collagenase, acetylcholinesterase, and pancreatic lipase. The second-highest content of total polyphenols in the Purple basil extract indicates that other phenolic constituents detected by HPLC, besides rosmarinic acid, contribute to the inhibition of enzymatic activity. Dark Opal basil I was the only extract exhibiting a high percentage of ferulic acid. Ferulic acid is the most abundant hydroxycinnamic acid found in plant biomass. It is a promising phytochemical with strong antioxidant activity [56]. It also contains a broad spectrum of other attractive biological properties, including antimicrobial, anti-inflammatory [56,57], and neuroprotective effects. Methanolic extracts of *O. basilicum* and rosmarinic acid demonstrated neuroprotective effects [58] and showed good in vivo effects in mice [47]. Although the results from our research showed that the Dark Opal I basil extract did not exhibit a significant neuroprotective effect, it did show great potential for antilipidemic, antidiabetic, and antimicrobial effects.

Previously, the hydroethanolic extract of Purple basil was shown to have good antibacterial activities against all tested Gram-negative and Gram-positive bacteria, with MIC values of 0.1–0.15 mg/mL [59]. In another study [60], the MIC was also reached for this basil cultivar; however, this occurred at higher concentrations. In our study, in addition to Purple basil generating an MIC at 0.15 mg/mL, Dark Opal basil I also exhibited the MIC at a concentration of 0.15 mg/mL. These results indicate that several factors can impact the antimicrobial activities of basil cultivars, and precise conditions which lead to good antimicrobial activities should be determined in the future.

Our study is the first to involve the in vivo testing of basil ethanolic extracts on zebrafish embryos. Previously, embryotoxic testing on *D*. *rerio* was carried out only for basil essential oils [61] and water extracts of *Ocimum sanctum*, whose chemical composition (revealing mainly flavonoids, terpenoids, saponins, and tannins) could not be compared with our results [62]. On the other hand, studies on *D*. *rerio* embryos were reported for the polyphenol-rich extract of the medicinal plant *Antirhea borbonica* [63]. Acetonic and aqueous extracts of this plant revealed the major phenolic compounds to be quercetin, caffeic, and coumaric acid. Although ethanolic basil extracts in our research also contained these phenol constituents, the most prevalent phenolic compounds for our samples were rosmarinic and ferulic acid. This could explain why developmental abnormalities (such as scoliosis and pericardial edema) were recorded in the concentration range of 2.3–7.2 mg/mL for *A*. *borbonica* and 0.17–0.33 mg/mL for ethanolic basil extracts. These results indicate that the major phenolic compounds detected in basil extracts have a stronger effect on *Danio rerio* zebrafish embryos in comparison with the major phenolic compounds identified in *Antirhea borbonica* extracts.

## 4. Materials and Methods

### 4.1. Materials

Six plant samples of five different cultivars of *Ocimum* sp., namely Purple, Cinnamon, Dark Opal I, Lemon, Genovese, and Dark Opal II basil were collected from the collection of plants at the Faculty of Agriculture, University of Zagreb, Croatia. This collection aims to maintain, evaluate, regenerate, and document plant genetic resources from medicinal and aromatic plants for use in agricultural production and breeding programs [64]. A collection of basil plants has been established at the Faculty of Agriculture, University of Zagreb, Croatia. All six plant samples were harvested in June 2021.

The plant name of all species is “great basil”, except for Lemon basil, whose plant name is “hoary basil” and which is a hybrid between *O. americanum* and *O. basilicum* [65].

### 4.2. Chemicals

All chemicals used for the preparation of artificial water (AW)—CaCl_2_ × 2H_2_O, MgSO_4_ × 7H_2_O, NaHCO_3_, KCl, and MeOH—as well as the Collagenase Activity Colorimetric Assay Kit (Catalog No. MAK293) were obtained from Sigma-Aldrich (St. Louis, MO, USA). Acarbose, soluble starch, α-amylase from porcine pancreas, iodine–potassium iodide solution, sodium phosphate dibasic anhydrous, and sodium dihydrogen phosphate anhydrous were all purchased from Sigma-Aldrich (St. Louis, MO, USA), whereas sodium chloride was purchased from Alkaloid (Skopje, North Macedonia) and HCl from Gram-mol d.o.o. (Zagreb, Croatia). All chemicals used in the neuroprotective assay were also purchased from Sigma-Aldrich: acetylcholine esterase (AChE), 5,5′-dithiobis-(2-nitrobenzoic acid) (DTNB), acetylcholine iodide (ACTI) and tacrine. Lipase from porcine pancreas, Type II, Orlistat, and lipase substrate, 4-nitrophenyl palmitate, and chloramphenicol were purchased from Sigma Aldrich (St. Louis, MO, USA), while the 1,4-dioxane for analysis was obtained from Merck Millipore (Burlington, MA, USA). Mueller Hinton agar used for the preparation of media used in antimicrobial activity testing was obtained from Merck (Darmstadt, Germany).

### 4.3. Preparation of Extracts

Extracts from six *Ocimum* sp. samples (Lemon basil, Dark Opal basil I, Dark Opal basil II, Genovese basil, Cinnamon basil, and Purple basil) were obtained using 70% ethanol according to the mass–volume ratios specified in Table 5. Prepared macerates were placed on shakers (120 rpm) for 48 h, after which they were filtered through filter paper and later evaporated on a rotary evaporator. The evaporated extracts were resuspended in methanol (33 mg/mL).

### 4.4. Headspace Solid-Phase Microextraction (HS-SPME)

HS-SPME was performed using an autosampler, PAL Auto Sampler System (PAL RSI 85, CTC Analytics AG, Zwingen, Switzerland). The extraction of the headspace VOCs was carried out on two SPME fibers of different polarities separately. Both fibers, one covered with DVB/Carbon WR/PDMS (divinylbenzene/carbon wide range/polydimethylsiloxane) and the other with PDMS/DVB (polydimethylsiloxane/divinylbenzene), were conditioned according to the manufacturer’s instructions prior to the extraction process. They were both purchased from Agilent Technologies (Palo Alto, Santa Clara, CA, USA). Prepared samples (1 g) were placed into 20 mL glass vials sealed with a polytetrafluorethylene (PTFE)/silicon septa stainless-steel cap. Equilibration of the sample was carried out at 60 °C for 15 min, after which the sample was extracted for 45 min. Thermal desorption of the fiber was executed directly to the GC column for 6 min at 250 °C.

### 4.5. Gas Chromatography–Mass Spectrometry Analysis of VOCs

A gas chromatograph (8890 Agilent Technologies, Palo Alto, Santa Clara, CA, USA) tandem mass-spectrometer detector (model 5977E MSD, Agilent Technologies) was used to analyze VOCs isolated from six basil samples. The separation of VOCs was underrun on a HP-5MS capillary column (30 m × 0.25 mm, 0.25 µm film thickness, Agilent Technologies, Palo Alto, Santa Clara, CA, USA). The GC–MS analysis conditions and the identification procedure of the compounds were as specified by Radman et al. (2022) [66].

### 4.6. Determination of Phenols Using HPLC

Identification and separation of polyphenolic compounds in the basil samples were performed on the Shimadzu LC-2010C HT HPLC system with a diode array detector at 280 nm. Standards and solvents used were HPLC-grade and formic acid (98–100%) was p.a. grade. The water used in the sample preparation, solutions, and analyses was double-distilled and purified using a Milli-Q water purification system by Millipore (Bedford, MA, USA). Standards dissolved in methanol were amentoflavone, epicatechin, ferulic acid, hydroxyphenyl-ethanol, caffeic acid, chlorogenic acid, naringenin, myricetin, rutin, rosmarinic acid, thymol, vanillic acid, quercetin, and coumaric acid; standards dissolved in DMSO were apigenin, luteolin, diosmetin, and diosmin. The concentration of each standard was 0.5 mg/mL.

The compounds were separated on an InertSustain C18 column (stationary phase particle size 5 μm, dimensions 250 × 4.6 mm) operated at 30 °C with the elution solvents A (0.1% formic acid) and B (methanol). The following method was used: 0–60 min gradient rinse with 90% of phase A to 90% of phase B; 60–65 min isocratic rinsing with 10% A and 90% B; 65–70 min isocratic rinsing with 90% A and 10% B. The total analysis time was 70 min with a flow rate of 0.5 mL min^−1^. The injected sample volume was 20 μL. Identification was determined by comparing the retention times and the UV spectrum of compounds in the sample with those of the corresponding standards.

### 4.7. Determination of Basil Extracts’ Inhibition Potential against Enzymatic Activities Using Spectrophotometric Assays

#### 4.7.1. Determination of Collagenase Inhibition by Basil Extracts Using a Spectrophotometric Assay

The in vitro collagenase inhibition assay was performed following the manufacturer’s instructions. Each *O. basilicum* extract was tested at 3.33 mg/mL. The 1,10-phenanthroline was used as a positive inhibitor. The rate of enzymatic hydrolysis of the (N-(3-[2-Furyl]acryloyl]-Leu-Gly-Pro-Ala) (FALGPA) substrate was monitored by its absorbance change at 345 nm (Infinite 200 PRO spectrophotofluorimeter, Tecan, Austria). All the reactions were performed in triplicate.

#### 4.7.2. Determination of Acetylcholinesterase Inhibition by Basil Extracts Using a Spectrophotometric Assay

The anti-acetylcholinesterase (anti-AChE) activity of basil extracts was measured using a microplate assay based on the Ellman method, as described previously [67]. Basil extracts (20 μL) at different concentrations (3.3 mg/mL, 1.65 mg/mL, 8.825 mg/mL) were added to the reaction mixtures. Hydrolysis of acetylthiocholine was monitored for 20 min by measuring the absorbance at 412 nm in the Tecan Infinite M200 Pro microplate reader. Tacrine, 100 nM (Sigma Aldrich, ≥99%), was used as a positive control for inhibitory activity. All reactions were performed in triplicate.

#### 4.7.3. Determination of Pancreatic Lipase Inhibition by Basil Extracts Using a Spectrophotometric Assay

The inhibitory potential of basil extracts on pancreatic lipase activity was determined by a colorimetric assay as previously described [68], but with a minor modification to adapt it to 96 well-plates. Porcine pancreatic lipase type II (Sigma Aldrich, L3126-25G) was suspended in Tris-HCl buffer (2.5 mmol, pH 7,4 with 2,5 mmol NaCl) at a concentration of 200 units/mL. *p*-Nitrophenyl palmitate (PNPP), a PL substrate, was dissolved in Tris-Na deoxycholate buffer (50 mM Tris-HCl pH 8, 5 mM Na deoxycholate) to a final concentration of 320 µM. Each tested extract was preincubated with PL for at least 10 min at 37 °C before adding the substrate mixture to begin the reaction, which was also maintained at 37 °C. By comparing the lipase activity of PL with and without the extract, the percentage of PL’s residual activity was calculated for each extract. Orlistat, a well-known PL inhibitor, served as the positive control. The solvent’s final concentration was fixed and did not increase beyond 5% [69].

#### 4.7.4. Determination of α-Amylase Inhibition by Basil Extracts Using a Spectrophotometric Assay

The antidiabetic properties of basil extracts were determined by the Caraway–Somogyi method [70,71] with minor modifications. Acarbose, also known as an α-amylase inhibitor, was used as a standard. In a 96-well microplate, 25 μL of basil extract or acarbose of a certain concentration and 50 μL of 0.5 mg/mL α-amylase solution prepared in 20 mM phosphate buffer (pH 6.9 with 0.006 M NaCl) were mixed and preincubated for 10 min at 37 °C. Afterwards, 50 μL of starch solution (0.05%) was added to initiate the reaction and the mixture was incubated for another 10 min at 37 °C. After the incubation, 25 μL of 1 M HCl was added to stop the reaction, followed by 100 μL of iodine–potassium iodide solution. A blank (sample without α-amylase), a positive control (only solvent with α-amylase), and a negative control (only solvent without α-amylase) were also prepared, and the reactions were performed using the protocol described above.

Absorbances of the samples (As), blanks (Ab), and positive (AC+) and negative (AC−) controls were read at 630 nm.

### 4.8. Determination of Antimicrobial Activity

The antibacterial activities of the basil extracts were evaluated using the broth microdilution test (CLSI standard M07. Wayne, PA: Clinical and Laboratory Standards Institute; 2018) The broth microdilution method is used to determine the lowest concentration of the assayed antimicrobial agent (minimal inhibitory concentration, MIC) that, under defined test conditions, inhibits the visible growth of the bacterium being investigated [72].

Basil extracts were tested in a concentration range of 0.5–300 μg/mL (*w*/*v*). Gram-positive bacteria (*Bacillus subtilis* ATCC 6633 and *Staphylococcus aureus* ATCC 6538), Gram-negative bacteria (*Pseudomonas aeruginosa* NCTC 12903, *Escherichia coli* NCTC 12241, *Klebsiella pneumoniae* ATCC 700608), and yeast *Candida albicans* ATCC 90028 were included in the bacterial panel. Inoculated media without the tested sample were used as positive controls and sterile media as negative controls, while chloramphenicol was used for quality control. The tests were carried out in an aerobic environment at 35 °C. Results were evaluated visually and with a spectrophotometer at a wavelength of 600 nm. If an MIC was detected, the result was confirmed by plating out the contents of the well.

### 4.9. In Vitro Antioxidant Activity Determination

Different dilutions of extracts were made from the stock solution (5 mg/mL) and used in all the methods. Four spectrophotometric methods were performed to evaluate antioxidant activity: the oxygen radical absorbance capacity (ORAC), reduction of radical cation (ABTS), 2,2-diphenyl-1-picrylhydrazyl-hydrate (DPPH), and ferric reducing antioxidant power (FRAP) assays. In order to determine total phenolic content, the Folin–Ciocalteu method was performed. Spectrophotometric measurements were performed in a 96-well plate using a microplate reader (Spectramax ABS plus, Molecular devices) in triplicate for all mentioned assays. The antioxidant activity of the extracts was tested by reactions with the appropriate reagents. After incubation, the change of color relative to the blank sample or control was measured, and the results were expressed as mean ± standard deviation (*n* = 3). A detailed description of the performed antioxidant activity assays can be found in our previously published research [73].

### 4.10. Zebrafish Embryotoxicity Test

Zebrafish *Danio rerio* females and males were kept under controlled laboratory conditions as described in [74]. Spawning was conducted following the procedure described by Babić et al. (2021) [75]. According to the OECD 236 protocol (2013), embryos (4–64 blastomeres; *n* = 30 per concentration) were transferred to 24-well plates (5 embryos per well; 2 mL of sample per well) and exposed to a range of concentrations spanning from 0.01 up to 0.33 mg/mL, prepared in serial dilutions. Artificial water was used as a negative control. After 96 h from the time of exposure, lethal and sublethal endpoints were measured as described in [75].

### 4.11. Statistical Data Processing

Statistical processing and graphical presentation of the obtained results tested spectrophotometrically were carried out using the statistical program GraphPad Prism 8.0 (GraphPad Software Inc., San Diego, CA, USA). The result of each enzymatic inhibition was expressed as the percentage of collagenase/acetylcholinesterase/α-amylase/pancreatic lipase inhibition (mean ± standard deviation (SD)), as well as data obtained from the in vitro antioxidant testing. Differences between the means were analyzed by Tukey’s one-way ANOVA test. Values of *p* < 0.05 and lower were considered significantly different. To correlate methods for antioxidant activity determination, Pearson’s correlation coefficient was used.

## 5. Conclusions

The variety of basil chemotypes from five different *O. basilicum* cultivars, all cultivated under the same geographic conditions and collected in June, was determined by implementing the extraction of their volatile compounds. Specific biological activities considering antioxidant and antimicrobial potential could be attributed to polyphenols as the secondary metabolites of the ethanolic basil extracts. The inhibitory potential against certain enzyme activities was also estimated by this research. All six basil extracts exhibited inhibitory potential against α-amylase activity. Purple basil, Dark Opal II and Lemon basil ethanolic extracts showed >50% inhibitory potential against pancreatic lipase. Collagenase activity was also inhibited by Purple basil and Dark Opal II. The observed difference in toxicity between the subspecies points to the need for the determination of the exact chemical composition in basil extracts. The findings from this study suggests that ethanolic basil extracts could contribute to the control of glucose absorption and consequently have the potential to be used as antiobesity and antidiabetic drugs [66] as well to be included in cosmetic products. Furthermore, these kinds of studies are necessary in order to evaluate the cultivars most suitable for complementary therapies.

## Figures and Tables

**Table 1:** Area percentages (%) of identified compounds by HS-SPME/GC-MS for the five basil cultivars. The percentages were obtained as mean values of triplicate GC-MS analyses.

Compound	Retention Index	Genovese Basil		Dark Opal Basil I		Dark Opal Basil II		Cinnamon Basil		Purple Basil		Lemon Basil	
		I	II	I	II	I	II	I	II	I	II	I	II
(Z)-Hex-3-en-1-ol	<900	-	-	-	-	-	-	0.47	0.81	-	-	0.28	0.30
α-Pinene	945	-	-	-	-	0.13	0.12	0.15	-	-	-	-	-
Benzaldehyde	971	0.19	0.32	-	-	0.07	-	-	-	-	-	-	-
Sabinene	982	-	-	-	-	0.16	0.13	-	-	-	-	-	-
β-Pinene	986	0.30	-	-	-	0.35	0.28	-	-	-	-	-	-
6-Methylhept-5-en-2-one	992	-	-	-	-	-	-	0.60	1.18	-	-	0.49	0.65
β-Myrcene	996	0.36	0.25	0.26	-	0.70	0.42	-	-	0.15	-	-	-
Limonene	1037	0.20	-	0.18	-	0.16	0.16	0.28	0.21	0.54	0.50	-	-
1,8-Cineole	1040	2.91	1.98	1.73	1.38	4.12	3.86	0.28	0.20	-	-	-	-
5-Methyl-5-vinyldihydrofuran-2(3H)-one (Lavender lactone)	1049	-	-	-	-	0.08	-	-	-	-	-	-	-
(*E*)-β-Ocimene	1055	0.25	-	-	-	-	-	0.31	0.27	0.32	0.23	-	-
Benzyl alcohol	1043	0.53	0.40	1.08	1.50	0.21	0.59	0.54	0.59	0.60	0.77	0.41	0.38
*trans*-Linalool oxide	1079	-	-	-	-	-	-	0.61	0.36	-	-	-	-
Fenchone	1094	-	-	-	-	-	-	1.11	1.05	-	-	-	-
Linalool	1104	40.82	47.83	45.77	54.60	60.43	65.20	8.22	9.16	24.75	28.82	0.35	0.21
6-Methyl-3,5-heptadien-2-one	1109	-	-	-	-	-	-	0.25	0.75	-	-	-	-
Hexyl propanoate	1110	-	-	-	-	-	-	-	-	0.05	-	-	-
2-Phenylethanol	1120	0.58	0.96	-	-	-	-	-	-	-	-	-	-
Camphor	1152	-	-	0.83	0.77	-	-	-	-	0.10	0.05	-	-
3,7-Dimethylocta-3,6-dienal	1188	-	-	-	-	-	-	0.70	0.48	-	-	1.22	1.25
α-Terpineol	1196	0.77	0.75	0.63	0.95	0.49	0.50	1.06	1.28	0.24	-	-	-
Estragole	1219	-	-	-	-	0.15	-	0.52	0.67	35.28	33.70	0.15	-
Octyl acetate	1217	0.32	0.10	-	-	-	-	1.07	1.12	0.17	0.15	0.94	0.56
Fenchyl acetate	1225	-	-	-	-	0.28	0.23	-	-	-	-	-	-
Nerol	1235	-	-	-	-	-	-	1.62	1.71	-	-	2.45	1.59
Neral	1248	-	-	-	-	-	-	13.12	12.13	-	-	20.57	25.54
Geraniol	1262	0.38	0.30	0.14	0.10	0.96	0.77	0.35	0.36	-	-	1.29	0.75
Citral	1276	-	-	-	-	-	-	24.26	22.77	-	-	36.27	41.47
Bornyl acetate	1290	1.22	1.07	0.99	0.95	-	-	-	-	-	-	-	-
Eugenol	1364	21.16	18.26	21.14	22.53	1.64	1.13	-	-	11.00	11.17	-	-
α-Copaene	1381	0.38	0.36	-	-	0.36	0.32	1.74	1.40	0.32	0.28	1.11	0.88
Sativene	1393	0.24	-	-	-	0.60	0.23	0.16	-	0.17		0.37	0.28
β-Elemene	1396	2.26	2.66	2.40	1.92	4.78	4.36	-	-	3.23	2.88	-	-
*cis*-α-Bergamotene	1432	3.46	2.44	3.04	2.80	-	-	-	-	-	-	-	-
*trans*-Caryophyllene	1424	-	-	-	-	1.59	1.49	4.52	4.64	1.51	1.43	5.23	4.00
α-Humulene	1459	0.54	0.53	0.48	0.05	0.51	0.45	0.79	0.78	0.92	0.78	-	-
*trans*-α-Bergamotene	1441	3.46	2.44	-	-	1.24	1.15	6.07	6.01	-	-	3.10	2.51
α-Guaiene	1444	1.01	1.16	1.11	1.01	2.19	2.02	0.49	0.55	1.40	1.31	-	-
Bicyclosesquiphellandrene	1648	-	-	-	-	-	-	-	-	0.92	0.24	1.08	0.88
(Z)-β-Farnesene	1463	-	-	-	-	0.16	-	-	-	-	-	0.67	0.48
Germacrene D	1485	3.79	4.21	1.48	1.10	3.94	3.41	0.92	0.93	4.62	4.03	2.15	1.09
β-Eudesmene	1490	-	-	-	-	-	-	1.11	1.07	-	-	-	-
Bicyclogermacrene	1501	1.04	0.92	0.67	0.10	2.90	2.48	-	-	1.50	1.15	-	-
δ-Guaiene	1510	2.15	2.51	2.22	1.98	4.75	4.31	-	-	3.10	2.98	-	-
β-Bisabolene	1513	-	-	-	-	-	-	0.73	0.64	-	-	0.37	0.30
γ-Cadinene	1518	3.78	3.55	3.50	3.00	1.58	1.47	-	-	1.91	1.79	-	-
*cis*-Calamenene	1528	0.52	-	0.60	-	0.35	0.26	-	-	0.33	-	-	-
Dihydroactinidiolide	1534	-	-	0.18	-	-	-	-	-	0.18	0.23	0.18	0.20
(*E*)-α-Bisabolene	1549	-	-	-	-	-	-	7.69	8.23	-	-	6.38	5.26
Caryophyllene oxide	1586	-	-	-	-	-	-	5.36	5.17	-	-	1.93	1.47
t-Cadinol	1647	2.80	3.80	4.55	3.82	0.90	0.97	-	--	1.93	1.67	-	-

**Table 2:** Area percentages (%) of identified polyphenolic compounds by HPLC.

Compound	RT	Area Percentage (%)					
		Genovese Basil	Dark Opal Basil I	Dark Opal Basil II	Cinnamon Basil	Purple Basil	Lemon Basil
Hydroxyphenylethanol	25.142	-	0.524	-	-	-	-
Chlorogenic acid	27.208	-	-	0.405	0.406	-	-
Epicatechin	29.250	0.343	0.500		0.241	-	0.447
Vanillic acid	30.383	-	-	3.054	3.439	3.451	-
Caffeic acid	30.625	3.522	3.185	-	-	-	2.744
Coumaric acid	36.108	-	1.039	2.677	-	0.552	-
Ferulic acid	37.158	-	16.842	-	1.027	1.444	-
Rosmarinic acid	40.025	13.422	-	17.096	21.001	18.117	24.826
Rutin	40.725	3.077	-	3.406	3.789	3.740	2.018
Diosmin	42.292	3.473	2.488	2.085	1.208	0.784	1.049
Myricetin	43.567	-	2.036	1.139	0.762	0.839	1.373
Naringenin	48.200	0.420	0.527	1.143	0.801	0.660	0.593
Quercetin	48.575	0.678	0.333	-	0.576	1.194	0.510
Luteolin	50.383	0.222	0.464	0.254	1.354	1.909	-
Thymol	58.658	0.120	0.089	-	0.331	-	0.242
Amentoflavone	59.067	0.017	0.021	0.020	0.089	0.221	0.067
Totals		25.293	28.048	31.279	35.025	32.911	33.869

**Table 3:** Antimicrobial effects (MIC and MBC µg/mL) of basil extracts.

	*S. aureus*	*B. subtilis*	*E. coli*
Purple basil	MIC 150 MBC 300	MIC 300	Partial inhibition 300
Dark Opal basil I	MIC 150 MBC 300	MIC 300	Partial inhibition 300

MIC—Minimum Inhibitory Concentration; lowest concentration of an antibacterial agent which completely prevents visible growth of the test strain of an organism (https://doi.org/10.1111/j.1469-0691.1998.tb00061.x). MBC—Minimum Bactericidal Concentration; minimum concentration of drug which kills 99.9% of test microorganisms in the original inoculum (https://doi.org/10.1016/B978-0-7236-0934-6.50018-5).

**Table 4:** Pearson’s correlation coefficients between the spectrophotometric methods used (DPPH, ABTS, FRAP, ORAC, and Folin–Ciocalteu method).

Correlation Coefficient (r)	DPPH	ABTS	FRAP	ORAC	Folin–Ciocalteu
**DPPH**	-				
**ABTS**	0.88 *	-			
**FRAP**	0.97 *	0.83 *	-		
**ORAC**	0.85 *	0.69	0.88 *	-	
**Folin–Ciocalteu**	0.84 *	0.59	0.82 *	0.95 *	-

* Correlation is significant (*p* < 0.05).

**Table 5:** Preparation of leaf macerates of selected basil species.

Sample Number	Sample	Mass (g)	Volume (mL)
*1*	Purple basil	15.51	200
*2*	Cinnamon basil	9.75	200
*3*	Dark Opal basil I	14.45	250
*4*	Lemon basil	10.97	183
*5*	Genovese basil	17.10	250
*6*	Dark Opal basil II	13.29	200

## Data Availability

Data is contained within the article.
